# Supplementary material for: Determinants of COVID-19 vaccine acceptance and hesitancy among adolescents and youths aged 10-35 years in sub-Saharan African countries: A systematic review and meta-analysis
Source: PLoS One. 2024 Oct 7;19(10):e0310827. doi: 10.1371/journal.pone.0310827 (PMC11458002; doi:10.1371/journal.pone.0310827)
Supplement: S1 Table — (DOCX) [file pone.0310827.s001.docx]

**S1 Table: Study characteristics included in the Systematic Review and Meta-analysis on the Prevalence of COVID-19 vaccine Acceptance, Hesitancy, and its determinants in sub-Saharan African countries**

| **Authors** | **Country** | **Adolescents** | **Year of Publication** | **Region** | **Sample Size** | **Prevalence acceptance (Event rate)** | **Prevalence Hesitance (Event rate** | **Study Design** | **Study setting** | **Determinants factors /Acceptance** | **Determinants of Hesitance** |
| --- | --- | --- | --- | --- | --- | --- | --- | --- | --- | --- | --- |
| Wang et al., 2022 [30] | Burkina Faso | Adolescents | 2022 | West Africa | 590 | 327 | 94 | Cross-Country | Community | To keep families safe from COVID-19 infection | Concerns about vaccine safety, and vaccine effectiveness. |
| Wang et al., 2022 [30] | Ethiopia | Adolescents | 2022 | East Africa | 542 | 292 | 92 | Cross-Country | Community | To keep self and family safe from COVID-19 infection based on Health worker's advice. | Concerns about the safety of the vaccine and effectiveness of the vaccine |
| Wang et al., 2022 [30] | Ghana | Adolescents | 2022 | West Africa | 300 | 300 | 136 | Cross-Country | Community | Among adolescents who were definitely or possibly accepting to receive COVID-19 vaccines for reasons keeping themselves and their families safe was the most common reason for vaccination | Concerns about the safety of the vaccine |
| Wang et al., 2022n [30] | Nigeria | Adolescents | 2022 | West Africa | 610 | 300 | 208 | Cross-Country | Community | Among adolescents who were definitely or possibly washing to receive COVID-19 vaccines, keeping themselves and their families safe was the most common reason for vaccination | Concerns about the safety of the vaccine and vaccine effectiveness |
| Wang et al., 2022 [30] | Tanzania | Adolescents | 2022 | East Africa | 620 | 104 | 210 | Cross-Country | Community | Among adolescents who were definitely or possibly accepting to receive COVID-19 vaccines, keeping themselves and their families safe was the most common reason for vaccination | Concerns about the safety of the vaccine (selected by 45% of participants), and concerns about the effectiveness of the vaccine (selected by 11% of participants). |
| Kyei-Arthu [31] | Ghana | Adolescents | 2022 | West Africa | 415 | 304 (73.3) | 110.8 | Cross section | Community | The education level is below the level of senior high school (Inverse). Previously received other vaccines. | Parents and guardians with Senior High School education were less likely (AOR = 0.390; CI 95% 0.153–  0.989; p = 0.047) to accept the COVID-19 vaccine for their children than those with less than  Senior High School education.  Inadequate information about the safety of COVID-19 vaccines and their adverse effects. Misinformation and religious beliefs, about COVID-19. physiological/natural immunity is  better than vaccine-induced immunity. |
| Mudenda et al., 2022 [32] | Zambia | Adolescents | 2022 | Southern Africa | 998 | 127 | 561 | Cross-section | Community | Overall, the odds of vaccine acceptance were higher among pupils who had higher knowledge scores (AOR = 11.75, 95% CI: 6.51–21.2), positive attitude scores (AOR = 9.85, 95% CI: 4.35–22.2), and those who knew a friend or relative who had died from COVID-19 (AOR = 3.27, 95% CI: 2.14–5.09). | The low vaccine acceptance among pupils is of public health concern, emphasising the need for heightened sensitisation programmes that promote vaccine acceptance among pupils in Zambia. |
| Faye et al., 2021 [33] | Bukinafaso | Adolencents | 2021 | West Africa | 506 | 96 | 152 | Cross section | Community | Perceived effectiveness and safety of COVID-19 vaccines increased the willingness to get vaccinated. Other factors increased willingness sociodemographic factors, such as sex, rural/urban residence, educational attainment and household composition (living with children and/or elderly), | Concerns about vaccine side effects, with the  highest levels of concern |
| Faye et al., 2021 [33] | Mali | Adolescents | 2021 | West Africa | 598 | 180 | 150 | Cross-Section | Community | Perceived effectiveness and safety of COVID-19 vaccines increased the willingness to get vaccinated. | Safety of COVID-19 vaccines and their adverse effects. Misinformation and religious beliefs, about COVID-19. physiological/natural immunity is  better than vaccine-induced immunity. |
| Faye et al., 2021 [33] | Senegal | Adolescents | 2021 | West Africa | 149 | 91 | 58 | Cross-section | Community | The perceived effectiveness of a vaccine to protect from COVID-19 and the safety of COVID-19 vaccines. The strongest association with vaccine safety was shown  for Senegal (PR=6.5; 95%CI 2.4 to 17.9), | The believe that COVID-19 vaccines is unsafe and concerned about vaccine side effects. |
| Faye et al., 2021 [33] | Sierra Leone | Adolescents | 2021 | West Africa | 428 | 179 | 18 | Cross-section | Community | The perceived effectiveness of a vaccine to protect from COVID-19 and the safety of COVID-19 vaccines increased the willingness to get vaccinated | The belief that the  vaccine has side effects or that the vaccine carries more risks  compared with routine vaccines lowers the willingness to accept vaccination. |
| Faye et al., 2021 [33] | Guinea | Adolescents | 2021 | West Africa | 561 | 56 | 309 | Cross-section | Community | the perceived effectiveness of a vaccine to protect from COVID-19 and the safety of COVID-19 vaccines increased the willingness to get vaccinated | The belief that the  vaccine has side effects or that the vaccine carries more risks  compared with routine vaccines lowers the willingness |
| Mustafa et al., 2023 [38] | DR Congo | Adolescents | 2023 | Central Africa | 522 | 172 | 351 | Cross-Section | Community | A higher level of  fear that “a member of my family” could contract COVID-19, a higher level of  perceived vulnerability to COVID-19 within the family, a higher level of  perceived susceptibility to COVID-19 within the family, and a history of vaccination against COVID-19 among parents. | The vaccine may not work and may give my child COVID-19 disease 212 60.4  The vaccine can give my child an illness other than COVID-19 146 41.6  The sting can be painful for my child. If it does not suit me, I will not have my child vaccinated. There could be side effects from the COVID-19 vaccine. I don’t believe in vaccines in general. I prefer my child to fight COVID-19 naturally, without using a vaccine. |
| Mudenda et al., 2022 [35] | Zambia | Young Adults | 2022 | Southern Africa | 326 | 78 | 248 | Cross-section | University | Male sex, being single and unemployed. Compared to females, male respondents were 86% more likely to accept the COVID-19 vaccine | Unemployed respondents were less likely to accept vaccination than their employed counterparts (aOR=0.32, 95% CI: 0.16-0.46). |
| Raja et al., 2022 [36] | Sudan | Young Adults | 2022 | West Africa | 220 | 121 | 96 | Cross section | University | Protect themselves and others from getting COVID-19. Factors associated with vaccine acceptance were history of COVID-19 infection (adjusted odds ratio (aOR) = 2. 2, 95% CI 1. 0–4.7, p = 0. 040), belief that vaccines are generally safe (aOR = 2.3, 95% CI 1. 2–4.5, p = 0.020), confidence that the vaccine can end the pandemic (aOR = 7.5, 95% CI 2. 5–22. 0, p<0.001), and receiving any vaccine in the past 5 years (aOR = 2.4, 95% CI 1.1–5.4, p = 0.031). | Concerns about vaccine safety and effectiveness were the main reasons reported by those who were hesitant. |
| Obi et al., 2022 [37] | Nigeria | Young Adults | 2022 | West Africa | 540 | 264 | 274 | Cross section | University students | The increase in the grade or year of study and the course being pursued (Medical students). | Cultural and religious beliefs the desire to wait and see the increased uptake of the vaccine. |
| Mustapha et al., 2021 [38] | Nigeria | Young Adults | 2021 | West Africa | 440 | 176 | 264 | Cross-section Studt | University students | Factors associated with acceptance of the vaccine were age of 25 years (adjusted odds ratio, aOR, 2.72; 95% confidence interval, CI, 1.44–5.16; p = 0.002), instructions from heads of institutions (aOR, 11.71; 95% CI, 5.91–23.20; p<0.001). | However, previous refusal to take vaccines (aOR, 0.18; 95% CI, 0.05–0.60; p = 0.006) was associated with decreased odds of accepting the COVID-19 vaccine |
| Berihun et al., 2022 [39] | Ethiopia | Young Adults | 2022 | East Africa | 442 | 226 | 216 | Cross-section | University students | Respondents who had a history of chronic medical illness (AOR: 4.340, 95%  CI: 1.166, 16.149), training on the COVID-19 vaccine (AOR: 4.755, 95% CI: 2.606, 6.674), history of regular vaccine uptake (AOR:  2.534, 95% CI: 1.412, 4.549), perception of COVID-19 severity (AOR: 4.109, 95% CI: 2.190, 7.710), the perception that COVID-19 can  be prevented by the vaccine (AOR: 2.420, 95% CI: 1.160, 5.049), and development of herd immunity against COVID-19 vaccines (AOR:  2.566, 95% CI: 1.431, 4.599) were factors significantly associated with COVID-19 vaccine acceptance. | Reasons for hesitancy include widespread  Pandemic conspiracy theories and a psychological need to comprehend numerous pandemic-related occurrences. As  a result, particularly in LMIC nations, there may be mistrust and unfavorable perceptions about the vaccine, a lack of  faith in the current healthcare system, and information shortages. |
| Aklil et al., 2022 [40] | Ethopia | Young Adults | 2022 | East Africa | 626 | 277 | 217 | Cross section | University students | The odds of having good knowledge among students who received health education | Moreover, those study participants whose fathers had attended primary education were 1.85 times more likely to have a positive attitude towards COVID-19 vaccination compared to study participants whose fathers were unable to read and write (AOR =1.85; 95% CI: 1.05, 3.24) |
| Potgieter et al., 2022 [41] | South Africa | Young Adults | 2022 | Southern Africa | 416 | 172 | 33 | Cross section | University students | Those who had comorbidity disease was 2.76 times higher as compared | Participants who had any doubts regarding the COVID-19 vaccine effectiveness and those who did not believe that face coverings and sanitation and physical distancing prevented the spread of COVID-19 had an increased odds of 12.62 (1.3–125.8), p = 0.031 and 6.76 (1.2–38.5), p = 0.031, of experiencing vaccine hesitancy. safety, efficacy and mistrust in the development |
| Orok E et al., 2022 [42] | Nigeria | Young Adults | 2022 | West Africa | 233 | 96 | 73 | Cross section | University students | Health workers recommendation to accept the vaccines while others reported that they would accept the vaccines  out of fear of contracting the virus and getting ill or spreading the virus to family and friends | The major reason for the rejection of the vaccines was lack of trust in the vaccine’s safety and efficacy of respondents also revealed that they lacked trust in the vaccine  source and in the government. Inadequate information regarding the adverse effects of  the vaccine, lack of trust in the vaccine source and in the  government, and belief that the vaccines would have future  medical implications. |
| Kanyike et al., 2021 [43] | Uganda | Young Adults | 2021 | East Africa | 600 | 224 | 376 | Cross section | University students | Factors associated with vaccine acceptability were being male (adjusted odds ratio (aOR) =  1.9, 95% CI 1.3–2.9, p=0.001) and being single (aOR= 2.1, 95% CI 1.1–3.9, p=0.022). Very high (aOR= 3.5, 95% CI 1.7–  6.9, p<0.001) or moderate (aOR =2.2, 95% CI 1.2–4.1, p=0.008) perceived risk of getting COVID-19 in the future,  receiving any vaccine in the past 5 years (aOR= 1.6, 95% CI 1.1–2.5, p=0.017), | The most given reason for hesitancy towards  the COVID-19 vaccine was concern  about its safety and side effects. having heard or  read negative information about the vaccine |
| Osur et al., 2022 [44] | Kenya | Young Adults | 2022 | East Africa | 665 | 279 | 386 | Cross-section | Community | Health information being shared openly. | Lack of information and concerns around vaccine safety and effectiveness were the main cause of COVID-19 vaccine hesitancy. Social media was the major source of information contributing to hesitancy. |

^AOR=Adjusted Odds Ratio, CR= Confidence Interval^
