# Supplementary material for: Determinants of COVID-19 vaccine acceptance and hesitancy among adolescents and youths aged 10-35 years in sub-Saharan African countries: A systematic review and meta-analysis
Source: PLoS One. 2024 Oct 7;19(10):e0310827. doi: 10.1371/journal.pone.0310827 (PMC11458002; doi:10.1371/journal.pone.0310827)
Supplement: Attachment — Submitted filename: Review comments.docx [file pone.0310827.s004.docx]

Thank you for giving me an opportunity to review this article about “Determinants of COVID-19 Vaccine Acceptance and Hesitance among Adolescents and Youths aged 10-35 years in Sub-Saharan African Countries: A Systematic Review and Meta-analysis.”

**Introduction**

It is not so critical as to why this study among adolescents and youth is essential? Is it a COVID-19 high risk group? Why should we worry about them? This could be fixed in the last paragraph

Looks like citations 17 to 20 in the second last paragraph have been manually inserted. Please rectify this.

In the statement, “As of February 2024, the percentage of COVID-19 vaccination in Africa was at 51.85…” is that a percentage?

**Methodology**

Though the inclusion criteria look fine, the authors just define some of the exclusion criteria as the opposite of inclusion which seems not right. I would advise the authors to use the PECO framework by Morgan RL (2018) to define eligibility.

Proof read the information sources section and remove any repetitions made.

The search strategy is not clear. Is what is being presented the full string that was used? And in which database was this used? Normally, a search is built in PubMed and modified for other databases. Additionally, it would be very useful to present the full search strategy indicating the strings as search in each of the databases together with the number of records and any filters added.

An important step of study selection is mixed-up; this makes following the article hard. I would advise the authors to be systematic following the five steps of systematic reviews clearly. Question, Search, Screening, Extraction, quality assessment, Data management. This flow would make readability easy.

Did you use Endnote, Rayyan of Covidence for screening? please be specific with this. Like I have noted above, please rearrange the write up for logical flow.

In the data analysis section, did you consider heterogeneity at 12 of 50%, or 75%. Please harmonize this.

**Results**

In the characteristics of included studies, it would be logical to state the reasons to why some studies were excluded after being subjected to full-text screening. this should also include how many studies were excluded for which reason.

It is also not clear the basis on which you performed the subgroup analysis. Please make this clear.

In Figure 1, please state the reasons for exclusion, not just reason 1 and reason 2

For verification purposes, please present a table for study characteristics indicating all the 23 studies, well cited with a column for quality of included studies.

Give keen attention to your P-values most especially in the subgroup analysis by region. First of all, it is a best practice to write P values with three digits after the decimal point (e.g. P=0.056), secondly, any p value that is displayed as 0.000 is written as p<0.001. finally, some of your p values have a greater than sign instead of less than sign, please rectify this.

In the determinants of vaccination acceptance section, I am still confused by the statement, “The odds of receiving Health Officers' information were 4.36 times more likely to influence acceptance of the COVID-19 vaccine than those who said no to the information from the health workers (AOR=4.36, 95%, CI, 2.28-8.32, I2=97.74, p < 0.001).” where the odds more likely? Or those who reported to have received the information were more likely? Please revise how you make these statement. The easiest way would have been, receiving information from the Health officer increased the odds of acceptance of the vaccine by more than 4-folds, or Receiving information from the health officer had a more than 4-folds increased odds of accepting the vaccine.

On your Tables, put a footnote to describe what AOR and CI mean.

**Discussion**

The first paragraph of your discussion seems to be more fitting into the introduction section.

The findings relating to the main outcome seem not well discussed. No adequate comparisons, no implications, I think you need to improve on this.
